# Development of an open-source soil water potential management system for horticultural applications, “*Open_Irr*”

**DOI:** 10.1016/j.ohx.2023.e00458

**Published:** 2023-07-20

**Authors:** Andrew M. Bierer

**Affiliations:** USDA-ARS, Appalachian Fruit Research Station, 2217 Wiltshire Rd. Kearneysville, WV 25430, United States

**Keywords:** Arduino, Irrigation, Soil moisture, LoRa radio, Open source, Control systems

## Abstract

Development of data driven, sensor-based irrigation has become possible with recent advancements in technology, yet due to a cost-barrier to entry, equitable adoption remains out of reach. Therefore, a simple microcontroller based LoRa platform, Open_Irr, was developed in the Arduino IDE to record resistive measures of soil matric potential and automate irrigation events. A single Open_Irr Node may connect with ≤ 16 Watermark® soil sensors and manage 4 irrigation groups, with Node sampling frequency, irrigation settings, and radio transmission settings being end-user adjustable without programming knowledge. Multiple Nodes may connect with a single Gateway to provide a scale-neutral solution for irrigation automation. A preliminary trial was conducted in spring 2022 to validate the performance of the Open_Irr platform in water deficit scenarios. Open_Irr recorded soil matric potentials in line with a commercial Watermark® sensor reader (n = 655); regression analysis yielded a coefficient of determination of 0.91 with Bland-Altman estimated fixed bias of ∼ 2 kPa. The Open_Irr platform was demonstrated to automate dripline irrigation events at several matric potential thresholds through connection to external solenoid valves. This demonstration directly conveys suitability for commercial and academic horticultural applications where automation of irrigation events or imposition of edaphic water stress is desired.

## Highlights

• An open-source soil matric potential management system was developed, Open_Irr.
• Constructed from commercially available hardware for ∼$250 USD.
• Scale-neutral irrigation automation by single unit or LoRa radio operation.
• Reads from ≤ 16 Watermark® matric potential sensors and temperature sensors.
• Commercial and research applications in climate-smart agriculture.

## Graphical abstract

**Figure:**
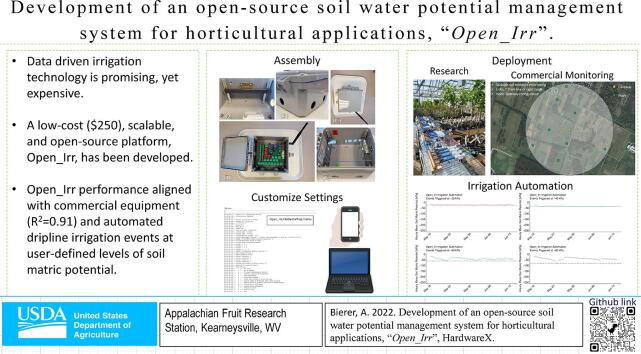

## Hardware in context

Horticultural drought stress is best measured through plant phenological measures of leaf and stem water potential, stomatal conductance, and photosynthetic efficiency [1]. However, physiological approaches have a cost barrier to widespread adoption through requirement of specialized equipment or lack of automation limiting scalability. In tree-fruit orchards, growers manage water through generalized regional evapotranspiration tables, producer experience based upon water rights and availability, or through emerging decision support systems utilizing sensor technologies. In fall of 2021, an effort to leverage new technology to improve and automate data collection of soil water status for research and commercial applications was initiated. The objective of this endeavor was to produce a low-cost and open-source datalogging platform to screen for drought tolerance within specialty cropping systems and implement responsive, *climate-smart*, irrigation programs in commercial operations.

The Open_Irr platform, phonetically *open ear*, is a microcontroller based datalogger programmed with a derivative of the C++ language, the Arduino individual development environment (IDE), for measurement, logging, and management of soil matric potential through irrigation automation. Open_Irr leverages an array of commercially available microcontroller modules and Watermark200ss soil matric potential sensors [2] for this task. A customized printed circuit board (PCB) was configured to integrate commercial hardware and placed in an environmental enclosure for field-use. Open_Irr can be used to output a low-level signal (3.3 V) in response to changes in soil matric potential with the intended use case of triggering an irrigation event. The Electronically Erasable Programmable Read Only Memory (EEPROM) on the Moteino-Mega USB [3] microcontroller enables end-user configuration of the Open_Irr device by saving a configuration structure altered in a simple menu interface during connection with a Serial terminal monitoring application. Open_Irr incorporates a 915 MHz long-range (LoRa) radio transceiver and may operate in a point-to-point Node-Gateway configuration with the Gateway capable of compiling data from multiple nodes. The Gateway also possesses the capability to output a low-level (3.3 V) signal based upon Watermark readings from multiple connected Nodes. Open_Irr platform specifications are provided in Table 1.

**Table 1:** Specifications table.

Hardware name	Open_Irr: soil water tension management system
Subject area	Environmental, planetary, and agricultural sciences
Hardware type	Field measurements and sensors
Closest commercial analog	900 M Monitor / IrroMesh https://www.irrometer.com/loggers.html
Open-source license	MIT
Cost of hardware	Single Node or Gateway: $250.00 (USD)
Source file repository	Github: https://github.com/andrewbierer/Open_IrrMendeley Data: https://doi.org/10.17632/sbzxm5rydj.1

The LoRa functionality can be described as a proprietary radio communication technology developed by Cycelo (patent 9647718-B2) that encodes information using the Chirp Spread Spectrum technique to transmit data on radio waves with lower power requirements than Bluetooth® or cellular options. The LoRa functionality utilizes < 1 GHz radio frequencies that do not require a license to enable “free” data transfer at moderate ranges; transmission of several kilometers is possible under ideal conditions. For additional information regarding the LoRa communication protocol, we direct readers to a summary provided by “The Things Network” [4]. The Open_Irr platform as described in this manuscript utilizes a RFM95_LoRa transceiver operating at 915 MHz as Open_Irr was developed in the United States. Users constructing Open_Irr units in other countries must check local regulations to identify the permitted frequencies and registration requirements; we direct readers to “The Things Network” aggregated list for more information [5]. All design files required for construction of Open_Irr units are provided in Table 2.

**Table 2:** Design files summary.

**Design file name**	**File type**	**Open-source license**	**Location of the file**
Open_Irr_Node.ino	Arduino code	MIT	https://doi.org/10.17632/sbzxm5rydj.1
Open_Irr_Gateway.ino	Arduino code	MIT	https://doi.org/10.17632/sbzxm5rydj.1
Gerber_HardwareX_Open_Irr_Node_PCB.zip	.zip	MIT	https://doi.org/10.17632/sbzxm5rydj.1
Gerber_HardwareX_Open_Irr_Gateway_PCB.zip	.zip	MIT	https://doi.org/10.17632/sbzxm5rydj.1
Schematic_HardwareX_Open_Irr_Node.pdf	.pdf	MIT	https://doi.org/10.17632/sbzxm5rydj.1
Schematic_HardwareX_Open_Irr_Gateway.pdf	.pdf	MIT	https://doi.org/10.17632/sbzxm5rydj.1

The most similar open-source hardware to Open_Irr is the ArduinoSoilH2O platform [6] which leverages similar hardware for datalogging from several commercial time-domain reflectometry soil moisture sensors. This platform was developed and is currently utilized by the USDA-ARS for research applications [7]. Several proprietary platforms possessing portions of comparable functionality have also been developed. The Irrometer® 900 M Monitor [8] is a battery-operated data-logger that records up to 8 Watermark sensors with user-addressable frequency and a small LCD display to visualize measurements. The Irrometer® Irromesh platform leverages a Node-Gateway configuration to read 3 Watermark sensors from each Node; the Gateway compiles and cellularly transmits data for cloud-based retrieval. The Monnit® ALTA platform leverages a single Watermark sensor in each package with cellular connectivity for retrieval and visualization. Campbell Scientific® provides a suite of datalogging accessories and multiplexors suitable to match functionality if comparable programming is implied. We are unaware of a platform that readily provides all functionalities of the Open_Irr system. A bill of materials for construction of Open_Irr Node and Gateway units has been provided in Table 3.

**Table 3:** Bill of materials for Open_Irr Node and Gateway units.

**Item number**	**Component**	**Number required**	**Cost unit^−1^ [USD]**	**Total cost [USD]**	**Source of materials**	**Material type**
*Open_Irr Node*						
1	Moteino-Mega USB, 915 MHz LoRa transceiver	1	44.95	44.95	Lowpowerlab.com	Semiconductor
2	DS3231 RTC module	1	17.5	17.5	Adafruit.com	Semiconductor
3	MicroSD card module	1	7.5	7.5	Adafruit.com	Semiconductor
4	MicroSD card, 8 GB	1	9.95	9.95	Adafruit.com	Semiconductor
5	Multiplexor, 16-channel CD74HC4067	2	5.95	11.9	Sparkfun.com	Semiconductor
6	Duckbill antenna	1	9.95	9.95	Sparkfun.com	Other
7	U.FL to SMA pigtail	1	8.95	8.95	Sparkfun.com	Other
8	U.FL connector	1	0.95	0.95	Lowpowerlab.com	Other
9	Battery cradle	1	2.99	2.99	Amazon.com	Other
10	Battery NiMH AA	6	1.29	7.74	Amazon.com	Other
11	FQP30N06 MOSFET	1	1.51	1.51	Digi-Key.com	Semiconductor
12	3-pin switch	1	0.95	0.95	Adafruit.com	Other
13	Resistor, 10 kΩ	3	0.03	0.09	Adafruit.com	Semiconductor
14	Resistor, 4.7 kΩ	2	0.03	0.06	Adafruit.com	Semiconductor
15	Rectifier diode, 1 N4007	2	0.05	0.1	Amazon.com	Semiconductor
16	Printed circuit board	1	2.04	2.04	Jclpcb.com	Other
17	Terminal block connector, 2-pin 5.08 mm pitch	17	0.1	1.7	Amazon.com	Other
18	Terminal block connector, 3-pin 5.08 mm pitch	16	0.9	14.4	Amazon.com	Other
19	Terminal block connector, 4-pin 5.08 mm pitch	1	1	1	Amazon.com	Other
20	Environmental enclosure	1	80.46	80.46	Enclosurehub.com	Composite
21	Plastic stock	1	5.94	5.94	McMaster-Carr.com	Polyvinyl chloride
22	Polycarbonate sheet	1	4.92	4.92	McMaster-Carr.com	Polycarbonate
23	Nylon spacers, 1.5″ x 3/8″ outer diameter	4	0.35	1.40	McMaster-Carr.com	Composite
24	Socket head screw, 10–32 2.5″	4	0.55	2.2	McMaster-Carr.com	Metal
25	Brass hinge, 2″ x 19/32″	1	4.77	4.77	Mc-Master-Carr.com	Metal
26	Screws, #6–32 × 1/2″	6	0.09	0.54	Lowes.com	Metal
27	Screws, #8–32 × 1/2″	2	0.17	0.34	Homedepot.com	Metal
28	Screw, #6 × 1/2″	2	0.01	0.02	Homedepot.com	Metal
29	Cable Grommet, 3/4″	3	1.01	3.03	Amazon.com	Other
	**Total cost per unit**			**247.85**		
*Open_Irr Gateway*						
1	Moteino-Mega USB, 915 MHz LoRa transceiver	1	44.95	44.95	Lowpowerlab.com	Semiconductor
2	DS3231 RTC module	1	17.5	17.5	Adafruit.com	Semiconductor
3	MicroSD card module	1	7.5	7.5	Adafruit.com	Semiconductor
4	MicroSD card, 8 GB	1	9.95	9.95	Adafruit.com	Semiconductor
5	Bluetooth module, HC-05	1	10.39	10.39	Amazon.com	Semiconductor
6	Duckbill antenna	1	9.95	9.95	Sparkfun.com	Other
7	U.FL to SMA pigtail	1	8.95	8.95	Sparkfun.com	Other
8	U.FL connector	1	0.95	0.95	Lowpowerlab.com	Other
9	Battery cradle	1	2.99	2.99	Amazon.com	Other
10	Battery NiMH AA	6	1.29	7.74	Amazon.com	Other
11	FQP30N06 MOSFET	1	1.51	1.51	Digi-Key.com	Semiconductor
12	3-pin switch	4	0.95	3.8	Adafruit.com	Other
13	Resistor, 10 kΩ	2	0.03	0.06	Adafruit.com	Semiconductor
14	Resistor, 4.7 kΩ	1	0.03	0.03	Adafruit.com	Semiconductor
15	Printed circuit board	1	1	1	Jclpcb.com	Other
16	Terminal block connector, 2-pin 5.08 mm pitch	1	0.1	0.1	Amazon.com	Other
17	Terminal block connector, 4-pin 5.08 mm pitch	1	1	1	Amazon.com	Other
18	Environmental enclosure	1	80.46	80.46	Enclosurehub.com	Composite
19	Plastic stock	1	5.94	5.94	McMaster-Carr.com	Polyvinyl chloride
20	Polycarbonate sheet	1	4.92	4.92	McMaster-Carr.com	Polycarbonate
21	Nylon spacers, 1.5″ x 3/8″ outer diameter	4	0.35	1.40	McMaster-Carr.com	Composite
22	Socket head screw, 10–32 2.5″	4	0.55	2.20	McMaster-Carr.com	Metal
23	Brass hinge, 2″ x 19/32″	1	4.77	4.77	Mc-Master-Carr.com	Metal
24	Screws, #6–32 × 1/2″	6	0.09	0.54	Lowes.com	Metal
25	Screws, #8–32 × 1/2″	2	0.17	0.34	Homedepot.com	Metal
26	Screw, #6 × 1/2″	2	0.01	0.02	Homedepot.com	Metal
27	Cable Grommet, 3/4″	3	1.01	3.03	Amazon.com	Other
	**Total cost per unit**			**231.99**		

## Hardware description

**Fig. 1:**
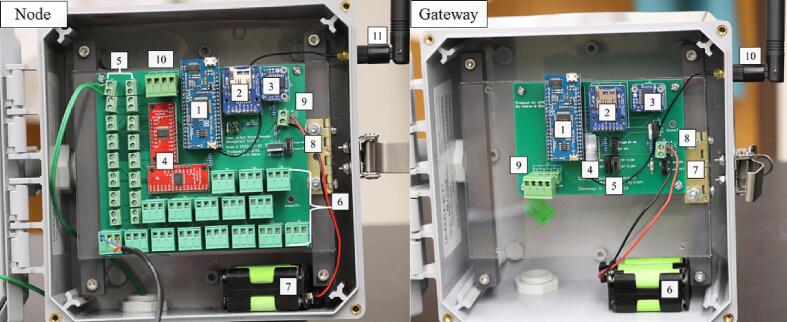
Open_Irr components **Node:** (1) Moteino-Mega USB; (2) MicroSD card module; (3) DS3231 real-time clock module; (4) CD74HC4067 16-channel multiplexor; (5) 2-pin terminal block screw connectors for Watermark200ss sensors; (6) 3-pin terminal block connectors for DS18b20 temperature sensors; (7) 6-NiMH-AA battery cradle; (8) On/off switch; (9) 2-pin terminal block screw connector for battery cradle; (10) 4-Pin terminal block connector for low-level output; (11) Radio antenna. **Gateway:** (1) Moteino-Mega USB; (2) MicroSD card module; (3) DS3231 real-time clock; (4) HC-05 Bluetooth module; (5) Bluetooth module control switches; (6) 6-NiMH-AA battery cradle; (7) On/off switch; (8) 2-pin terminal block screw connector for battery cradle; (9) 4-Pin terminal block connector for low-level output; (10) Radio antenna.

The Open_Irr platform is composed of “Node” and “Gateway” units to provide scalability but may be operated with or without a connected Gateway. Both the Node and Gateway are constructed utilizing the Moteino-Mega USB microcontroller board utilizing the ATMEL ATMEGA1284P processor; the 915 MHz LoRa transceiver was specified per the available “industrial/scientific/medical” frequency band in the originating region (USA). To facilitate an external radio antenna, U.FL connectors were soldered to the Moteino-Mega; 15 cm external U.FL to RP-SMA pigtails leading to 200 mm ½ wave 915 MHz duckbill antennas permitted external mounting on the environmental enclosure. Each unit contains a DS3231 real-time clock (RTC) module [9] with a CR1220 coin cell battery for time keeping, a MicroSD card module [10] with 8 GB MicroSD card for data storage, a 6-NiMH-AA battery cradle for wireless power, and a voltage divider circuit for battery life estimation (Fig. 1). A customized PCB was designed to facilitate ease of reproduction for each unit. The complete schematics, PCB files, and firmware can be found in the online Mendeley data repository [11] used for this publication, and the project’s GitHub page [12]. Microcontroller modules, terminal block connectors, 4.7 & 10 kΩ resistors, FQP30N06 MOSFETs, 3-pin switches, and 1 N4007 rectifier diodes were soldered into place upon receipt of PCBs with 60/40 lead rosin-core solder. Stacking headers were used on components where reuse was likely (Moteino-Mega USB, DS3231).

The Node device is configured with 2, 16-channel CD74HC4067 multiplexors [13] to enable ground isolation from sensor to sensor per the sensor manufacturer guidelines [14]. Sixteen 2-pin terminal block screw connectors allow connection of ≤ 16 Watermark sensors; the direction of the Watermark wires in the connection is inconsequential. Sixteen 3-pin terminal block screw connectors allow connection of ≤ 16 DS18b20 temperature sensors [15]. At the time of writing, temperature sensors are configured in a STAR topology; however, transition to a BUS topology is imminent to conform with manufacturer best practice specifications [16]. Temperature sensor connections from left to right on each are: GND, DATA, and VCC. A 4-pin terminal block screw connector is connected to digital output pins of the Moteino-Mega USB; these digital output pins (named left to right as pinouts/outputs 1,2,3,4) can be toggled to a HIGH (3.3 V) state in response to Watermark sensor readings as defined in the device’s configuration menu.

The Gateway device is configured with a 4-pin terminal block screw connector connected to digital output pins of the Moteino-Mega USB like in the Node; these digital output pins (named left to right as pinouts/outputs 1,2,3,4) can be toggled to a HIGH (3.3 V) state in response to Watermark sensor values from radio transmissions received by connected Open_Irr Nodes. The Open_Irr Gateway device has 3 additional 3-pin switches to toggle RX, TX, and VCC states of a HC-05 Bluetooth module with the intention of Bluetooth user interface in a future iteration. At the time of writing, interfacing with the gateway must be made through a Serial terminal monitoring application. The Gateway should be continuously powered using the Micro-USB port of the Moteino-Mega through an AC/DC power supply (3.3–16 V, 3.6–12 V recommended). Temporary power outages are circumvented by integration of a 6-NiMH-AA battery cradle as in the Node device.

The voltage divider circuit in either device was specified using 10 kΩ and 4.7 kΩ resistors and toggled using a FQP30N06 MOSFET to limit current drain. Digital pin 30 of the Moteino-Mega USB was defined as an output and connected to the gate of the MOSFET and a 10 kΩ pull-down resistor to ground; the MOSFET source pin was connected to the common ground. The MOSFET drain pin was connected sequentially to the 4.7 kΩ resistor, the analog pin 7 of the Moteino-Mega USB, the 10 kΩ resistor, and the battery VIN. Analog pin 7 on the Moteino-Mega was set as an input and used to read VIN from the battery cradle after digital pin 30 was set high to close the source-drain MOSFET circuit.

**Fig. 2:**
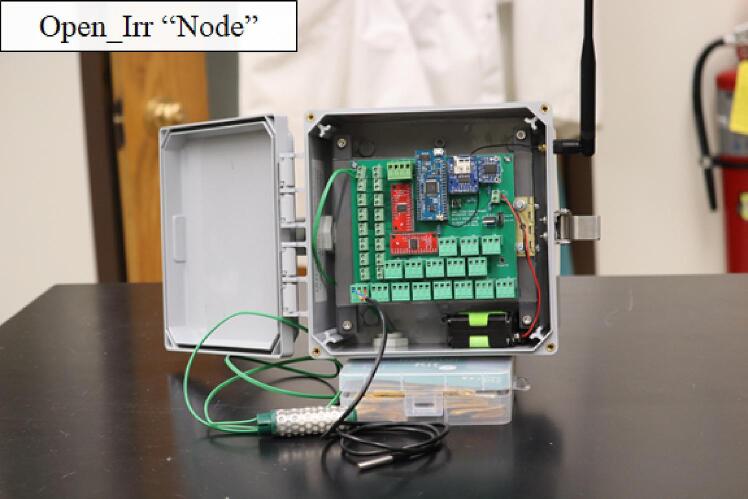
A complete Open_Irr Node unit inside the environmental enclosure. One Watermark sensor (green) and one temperature sensor (black) are connected. (For interpretation of the references to colour in this figure legend, the reader is referred to the web version of this article.)

A 6_L_-by-6 _W_-by-4_D_ inch locking latch IP68 rated environmental enclosure [17] was used to house the PCB, battery cradle, and mount the radio antenna (Fig. 2). The environmental enclosure listed provides brackets to allow mounting the enclosure to a flat surface. Therefore, a variety of final enclosure mounting configurations are possible. Final mounting solutions should take care to avoid detrimental positioning of the cable grommets, collecting rainfall if placed upwards or towards sprinkler heads, as examples. The additional consideration of avoiding radio antenna obstructions will better suit deployments utilizing Gateway and Node units.

• Datalogging for multiple Watermark sensors.
• Automation of data-driven irrigation events.
• Research applications in drought stress and imposition of water deficit.
• Controlled environment research applications.

## Design files summary

**Open_Irr_Node.ino:** Open_Irr Node program written in the Arduino IDE, uploaded to Moteino-Mega USB microcontroller in the Node unit. Should only be altered by advanced users.

**Open_Irr_Gateway.ino:** Open_Irr Gateway program written in the Arduino IDE, uploaded to Moteino-Mega USB microcontroller in the Gateway unit. Should only be altered by advanced users.

**Gerber_HardwareX_Open_Irr_Node_PCB.zip:** Open_Irr Node gerber zip file. Provide to PCB manufacturers to obtain PCB.

**Gerber_HardwareX_Open_Irr_Gateway_PCB.zip:** Open_Irr Gateway gerber zip file. Provide to PCB manufacturers to obtain PCB.

**Schematic_HardwareX_Open_Irr_Node.pdf:** Open_Irr Node electrical schematic of included components.

**Schematic_HardwareX_Open_Irr_Gateway.pdf:** Open_Irr Gateway electrical schematic of included components.

## Bill of materials summary

### Build instructions

#### Soldering the PCB

Soldering was performed by hand using a Hakko-FX888D-23BY soldering station with a T19-I sharp tip and temperature set at 343 °C. Printed circuit boards were ordered to ease construction; the required Gerber design files to provide to PCB manufacturers have been provided at the links above. After receipt of the PCB, components were soldered into place per the linked schematic and layouts in Fig. 1. All components are soldered so that they are accessible from the Silkscreen (side with text) side of the PCB. Resistors are non-polar components and orientation is inconsequential where utilized; resistor locations on the PCB are marked with their respective value (kΩ) in text on the Silkscreen layer.

**Fig. 3:**
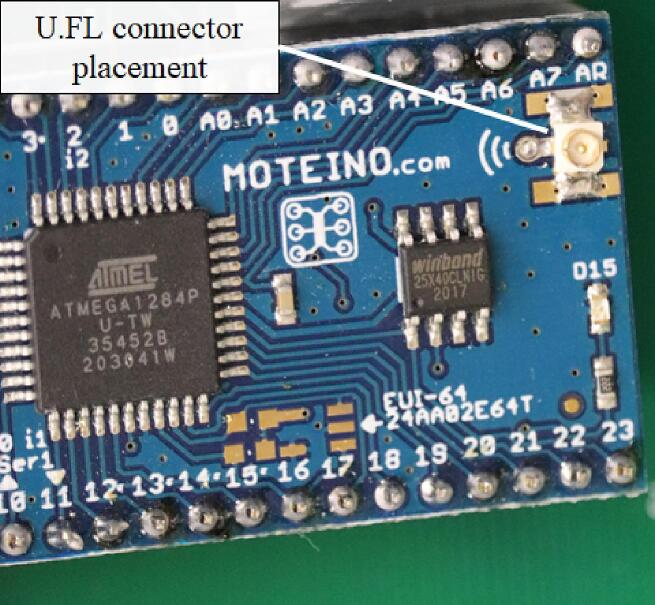
Placement of the U.FL connector on the Moteino-Mega USB module for later attachment of the radio antenna with a U.FL to SMA cable.
• Gateway
  o Identical to Node.

• Node
  o The Moteino-Mega USB must be soldered to the PCB so the Micro-USB connector faces up and is nearest the top edge of the PCB. However, before soldering the Moteino-Mega USB to the PCB, it is best to first solder the U.FL connector to the Moteino-Mega USB module. The U.FL connector must be soldered to the Moteino-Mega USB module so the wide surface mount wings of the U.FL connector attach to the wide surface mount platforms on the Moteino-Mega USB. Consequently, the narrow surface mount wing of the U.FL connector is soldered to the circular through hole for attaching a simple wire antenna on the Moteino-Mega USB module. The U.FL connector can be soldered to either side of the Moteino-Mega USB. Note: this is the most difficult component to solder – it is suggested to first place a small portion of solder on one of the wide surface mount platforms of the Moteino-Mega USB. Then, using forceps in one hand and the soldering iron in the other, reheat the solder and place the U.FL connector using forceps. The other surface mounts of the U.FL connector can now be carefully soldered (Fig. 3).

The DS3231 RTC uses I^2^C protocol and connected Serial Clock (SCL) and Serial Data (SDA) lines to digital pins 16 and 17 respectively on the Moteino-Mega USB; the Square Wave (SQW) line was connected to digital pin 24 of the Moteino-Mega USB to handle timer interrupts.

• Node
  o Solder the DS3231 module to the PCB so the integrated circuit faces up and the CR1220 battery cradle faces the PCB; the VIN pin will be on the top edge of the PCB and the RST pin will be closer to the bottom edge of the PCB.
• Gateway
  o Solder the DS3231 module to the PCB so the integrated circuit faces up and the CR1220 battery cradle faces the PCB; the VIN pin will be on the left-hand side and the RST pin will be on the right-hand side.

The MicroSD card module used SPI protocol and connected the Serial Clock (CLK) pin to digital pin 7 of the Moteino-Mega USB, Data Out (DO) pin to digital pin 6, Data In (DI) to digital pin 5, Chip Select (CS) pin to digital pin 28, and Chip Detect (CD) pin to digital pin 29.

• Node
  o Solder the MicroSD card module so all module components face up; the 5 V pin will be on the left-hand side and the CD pin will be on the right-hand side.
• Gateway
  o Identical to Node.

Each CD74HC4067 multiplexor was connected to ground through a rectifying diode. The Enable (EN) pins of each multiplexor connected to digital pin 22 of the Moteino-Mega USB, the Select 0,1,2, and 3 (S0,S1,S2,S3) pins of each multiplexor connected to digital pins 18,19,20, and 21, respectively. The Signal (SIG) pin of one multiplexor connected to digital pin 26 of the Moteino-Mega USB; the SIG pin of the other multiplexor connected to analog pin 1 and to digital pin 27 through a 10 kΩ resistor. Channels 0 to 15 of one multiplexor connected to one side of the 16, 2-pin terminal blocks while channels 0 to 15 of the other multiplexor connected to the opposing side of the 2-pin terminal blocks, respectively. Wiring of the multiplexors was designed in this way to conform to manufacturer reading instructions of Watermark sensors [14].

• Node
  o Solder each CD74HC4067 module so the integrated circuit faces up, in doing so there is only one way the modules fit on the PCB. Solder these 2-pin terminal block connectors so all openings face the left-hand side of the PCB. The two rectifying diodes are polar components and must be soldered so the stripe is nearest the bottom edge of the PCB.
• Gateway
  o Not applicable.

Power and ground from the battery cradle were routed to one side of a 2-pin terminal block. The powered side was connected to Voltage IN (VIN) 2 of the Moteino-Mega USB; the ground side was connected to one end of a 3-pin slide switch, the middle pin of the slide switch connected to the Ground (GND) 2 of the Moteino-Mega USB. The other end of the 3-pin slide was left unconnected to permit user toggle of Open_Irr battery power via the slide switch.

• Node
  o Solder the 2-pin terminal block connector so the openings face the right-hand side of the PCB. The direction of the 3-pin switch is inconsequential.
• Gateway
  o Identical to Node.

Power from the battery cradle was also routed to a voltage divider circuit consisting of 10 and 4.7 kΩ resistors with analog pin 7 of the Moteino-Mega USB connected to the junction point of the voltage divider circuit. The voltage divider circuit was made toggleable by connecting the ground end of the divider circuit to the drain pin of a FQP30N06 metal–oxide–semiconductor field-effect transistor (MOSFET). The source pin of the MOSFET was connected to the common ground while the gate pin was connected to digital pin 30 of the Moteino-Mega USB and a 10 kΩ pull-down resistor to ground. Solder the MOSFET commensurate with the Gate-Drain-Source pins, G-D-S, indicated on the Silkscreen layer.

• Node
  o Solder the MOSFET so the heat dissipation fin is nearest the bottom edge of the PCB.
• Gateway
  o Solder the MOSFET so the heat dissipation fin is nearest the left-hand side of the PCB.

Sixteen 3-pin terminal block connectors allow connection of 16 DS18B20 temperature sensors utilizing the Dallas 1-Wire protocol. These sensors have 3 leads in total (GND, Vin, DATA) and the 1-Wire protocol allowed routing a common data line to digital pin 1 of the Moteino-Mega USB; the data line was also connected to 3.3Vin through a 4.7 kΩ pull-up resistor.

**Fig. 4:**
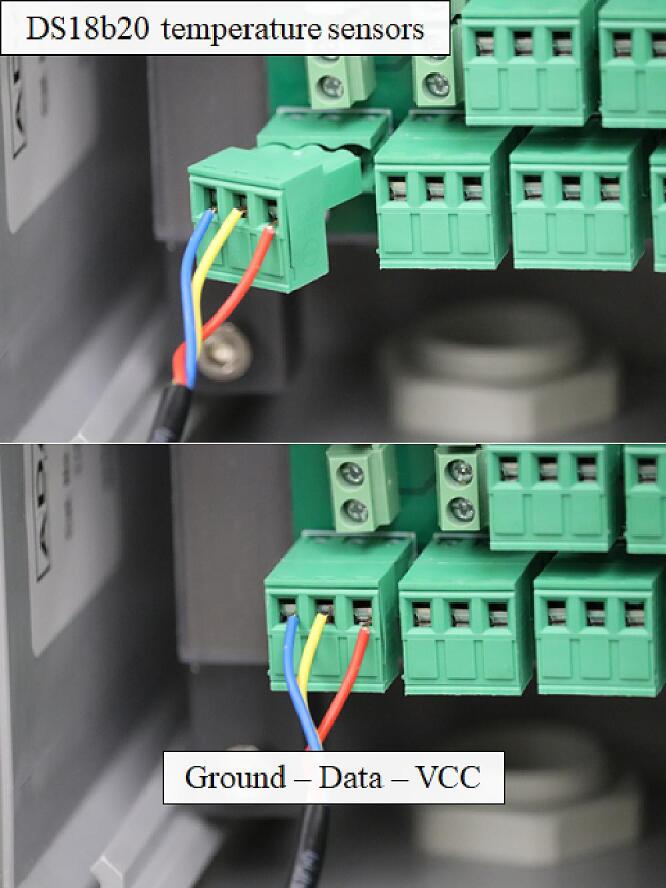
Correct placement of the male–female 3-pin terminal block screw connectors to Open_Irr Nodes. Wiring must be ground-data-vcc from left to right regardless of connector type.
• Gateway
  o Not applicable.

• Node
  o Solder the 3-pin terminal block connectors so the screw heads of the male portion of the connector are nearest the bottom of the PCB. From left to right, as plugged into the female header, the DS18B20 temperature sensor connections are GND-Data-VCC (Fig. 4).

One 4-pin terminal block connector allowed external wire connection, left to right, to digital pins 12, 13, 14, and 3 of the Moteino-Mega USB (19 to 22 on the Gateway), respectively. Firmware options are provided to set the pins HIGH based on soil matric potential readings. It is intended for this output to act as a signal enabling ancillary equipment, such as irrigation infrastructure, to function based on sensor readings made by Open_Irr Nodes.

• Node
  o Solder the 4-pin terminal block connector so the screw heads of the male portion of the connector are nearest the bottom of the PCB.
• Gateway
  o Identical to Node.

One HC-05 Bluetooth module and three 3-pin slide switches are included in the Gateway unit to permit future Bluetooth communication with the Open_Irr Gateway.

• Node
  o Not applicable
• Gateway
  o Although currently nonfunctional, the HC-05 Bluetooth module is soldered to the PCB so the EN pin is nearest the top edge of the PCB and the STATE pin is nearest the bottom edge of the PCB. Three additional 3-pin slide switches are soldered to the PCB which are intended to permit user toggle of the Bluetooth connection with the Open_Irr gateway, the direction of the switches is inconsequential.

### Preparing the enclosure

**Fig. 5:**
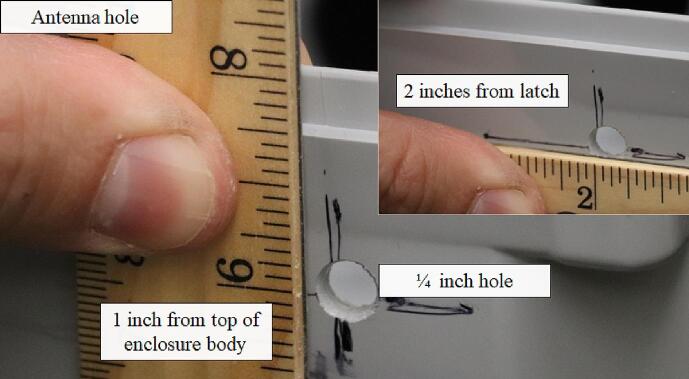
A ¼-inch hole must be drilled through the environmental enclosure to permit external placement of the radio antenna.

The environmental enclosure must be prepared to allow mounting the Open_Irr PCBs and installation of the radio antenna and cable grommets. A ¼-inch hole was drilled 2 in. to the right of the locking latch at a 1-inch depth relative to the enclosure body to facilitate antenna installation. Post construction and antenna attachment, the antenna hole was sealed with silicone to prevent water leaks (Fig. 5).

**Fig. 6:**
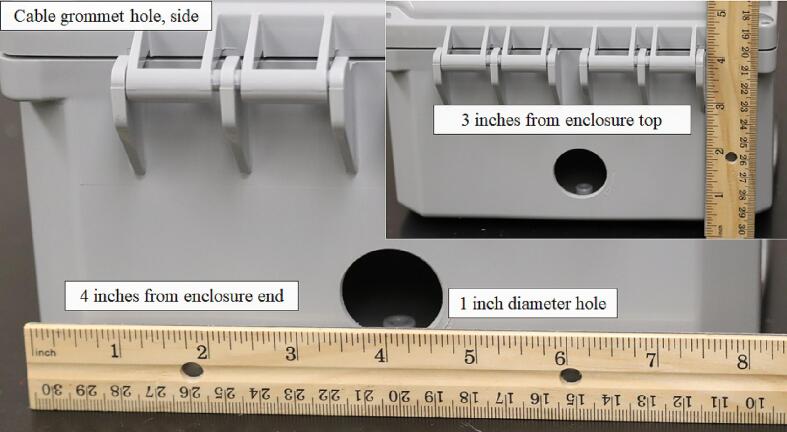
Placement of the 1-inch-diameter hole for installation of the ¾-inch cable grommet on the side of the environmental enclosure.

A 1-inch-diameter hole was cut on the left hand side at a 3-inch depth relative to the enclosure body and 4 in. from either length of the enclosure to install a ¾-inch cable grommet (Fig. 6).

**Fig. 7:**
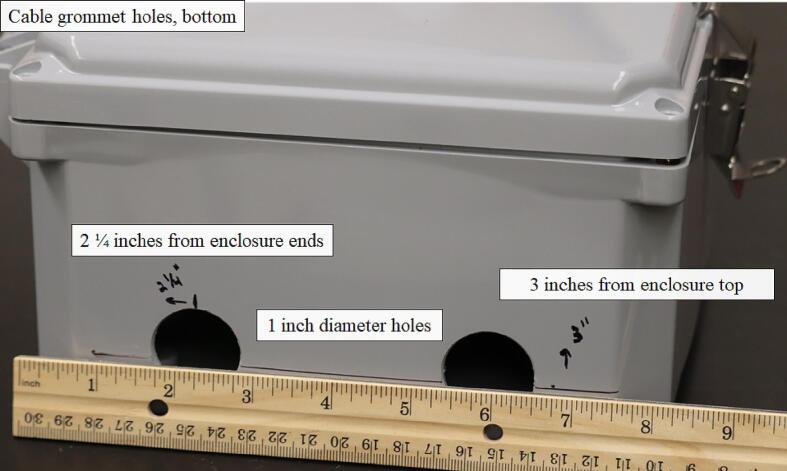
Placement of the 1-inch-diameter holes for installation of the ¾-inch cable grommets on the bottom of the environmental enclosure.

Similarly, two additional 1-inch-diameter holes were cut at 2 ¼ inches from each end of the width of the enclosure and a 3-inch depth relative to the enclosure body to accommodate two additional ¾-inch cable grommets (Fig. 7).

**Fig. 8:**
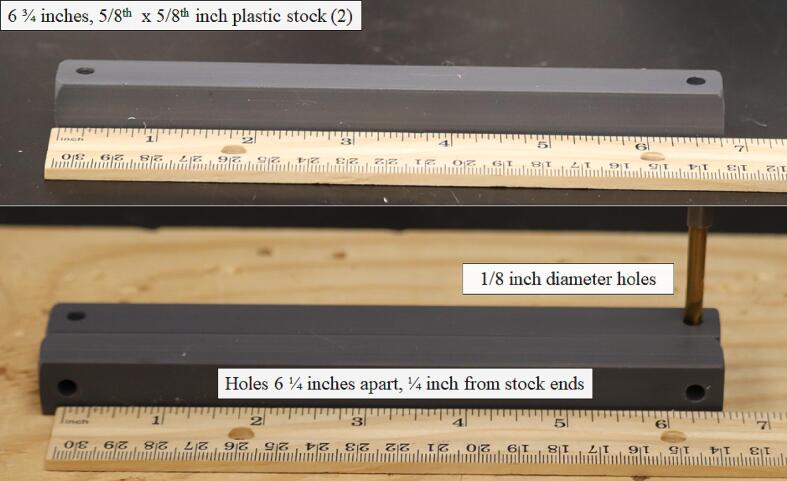
Preparation of the 5/8-inch by 5/8-inch plastic stock utilized in the mounting structure inside the environmental enclosure.

Inside the enclosure four 1 ½-inch by 3/8-inch (outer diameter) nylon spacers offset a support platform constructed from two 5/8-inch by 5/8-inch pieces of plastic stock cut to 6 ¾-inches in length. To prepare plastic stock for mounting inside the enclosure, drill two 1/8-inch-diameter holes ¼-inch from the long ends of the stock (Fig. 8).

**Fig. 9:**
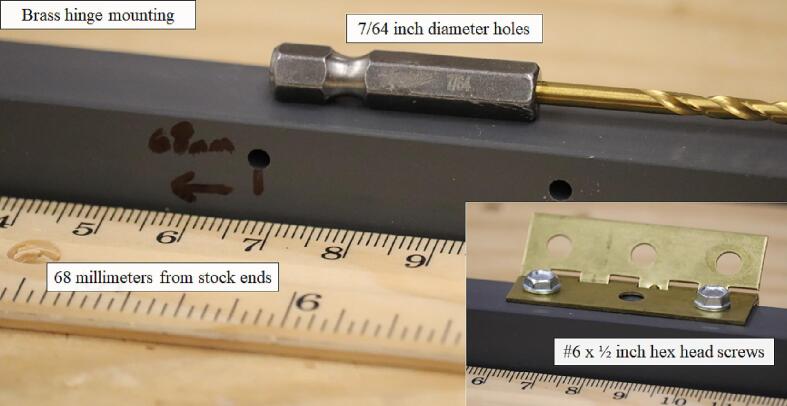
Placement of the holes for attaching the brass hinge to one of the pieces of plastic stock.

On the latch side of the enclosure, the plastic stock connects to a 2-inch brass ball-bearing hinge; to prepare the stock for hinge mounting, drill two 7/64-inch holes 68 mm proximal to either end of the stock (Fig. 9).

**Fig. 10:**
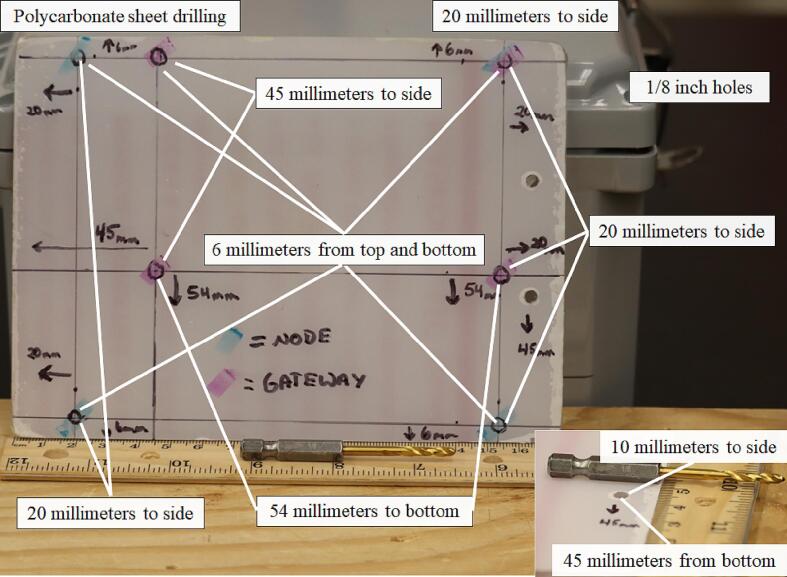
Preparation of the 5-inch by 7-inch polycarbonate sheet for attaching Open_Irr Nodes and Gateway units.

The hinge connects to the plastic stock via two ½-inch (#6) hex head screws. The other side of the hinge connects to a 5-inch by 7-inch polycarbonate sheet 1/8-inch thick with two ½-inch (#8–32) bolts and nuts. To prepare the polycarbonate sheet for mounting the hinge, drill two 1/8-inch-diameter holes 45 mm from the long edge and 10 mm proximal to the narrow ends of the sheet. The Open_Irr PCBs were designed with 1/8-inch diameter through-holes to permit mounting onto the polycarbonate sheet using ½-inch (#6–32) bolts and nuts. To prepare the polycarbonate sheet for Node mounting, drill four 1/8-inch-diameter holes 20 mm and 6 mm proximal to the sides of the polycarbonate sheet. To prepare the polycarbonate sheet for Gateway mounting, drill two 1/8-inch-diameter holes 20 mm and 45 mm proximal to the narrow ends of the polycarbonate sheet and 6 mm proximal to the top edge of the sheet. The bottom two holes for mounting the gateway are also positioned 20 mm and 45 mm proximal to the narrow ends of the polycarbonate sheet and 54 mm proximal from the bottom edge of the sheet (Fig. 10).

**Fig. 11:**
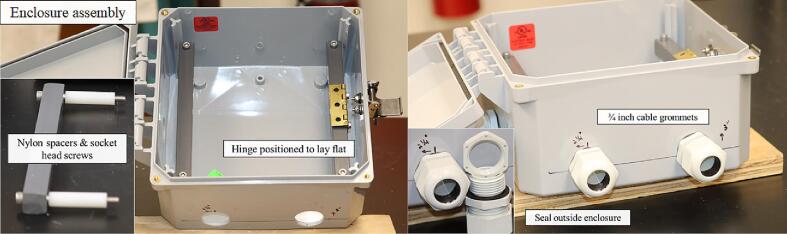
Placement of the mounting hardware inside the environmental enclosure and attachment of the cable grommets with the rubber gasket on the exterior of the enclosure.

After all drilling is completed, the mounting solution can be installed in the environmental enclosure. Place the 2 ½-inch socket head screws (#10–32) through each piece of plastic stock, placing the 1 ½-inch nylon spacers on the other side. Then, screw into place inside the four corners of the environmental enclosure, ensure the vacant side of the brass hinge faces upward and can lay flat (Fig. 11).

**Fig. 12:**
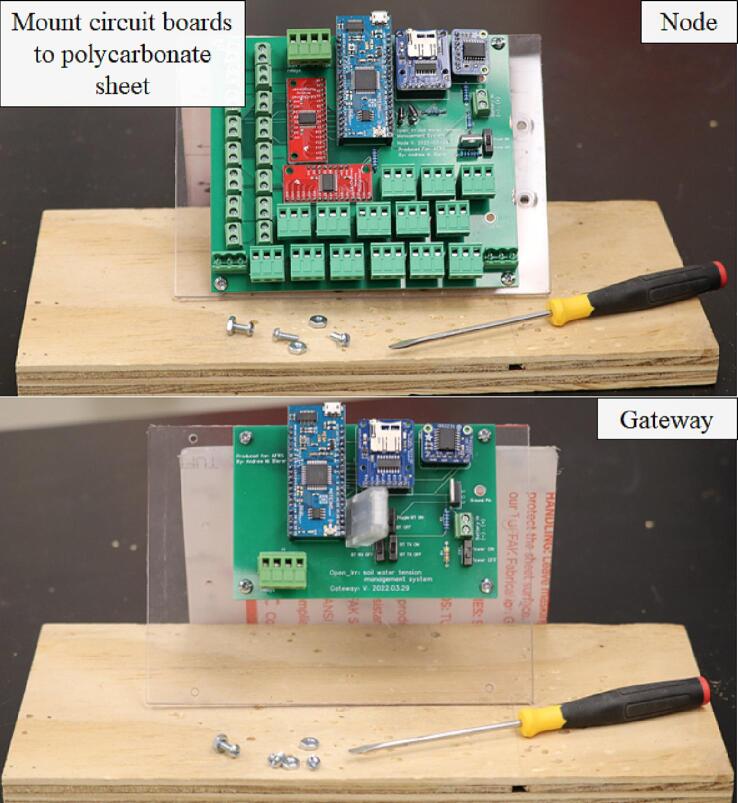
Mounting the fully soldered Open_Irr Node and Gateway PCBs to the polycarbonate sheet.

Install the ¾-inch cable grommets so the rubber gasket is on the exterior of the enclosure. If soldering of the PCB has been completed, mount the respective Node or Gateway PCB to the polycarbonate sheet using ½-inch screws and nuts (#6–32) (Fig. 12).

**Fig. 13:**
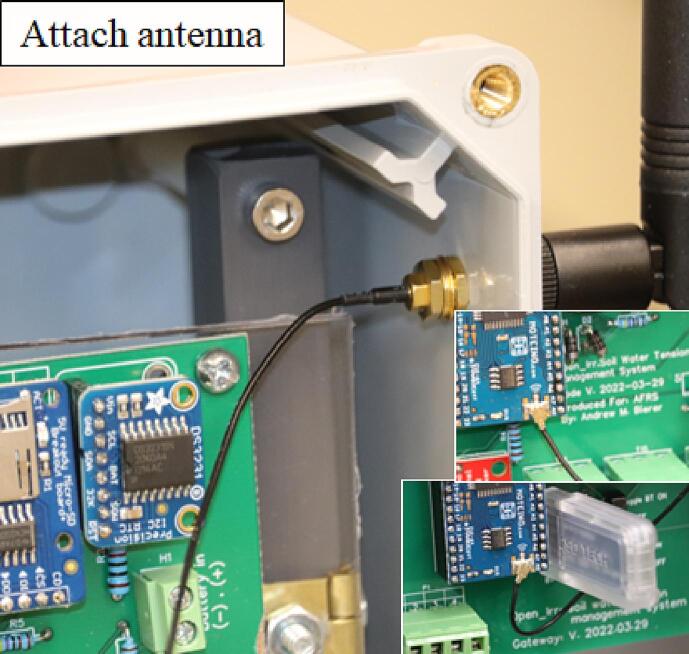
Attachment of the external radio antenna to the environmental enclosure and Open_Irr PCBs.

Install the polycarbonate sheet inside the enclosure by attaching it to the brass hinge using ½-inch screws and nuts (#8–32), then attach the radio antenna to the enclosure using the U.FL to SMA pigtail/connector. The U.FL connector on the pigtail can now be attached to the U.FL connector on the Moteino-Mega USB (Fig. 13).

## Operation instructions

### Open_Irr operation

Before operation of Open_Irr Node or Gateway units, users must download the required Serial terminal monitoring software to interface and communicate with the system. Due to the interactive menu interface of the Open_Irr system in the Serial terminal, additional action in a programming development environment after initial firmware upload is not necessary for typical operation. If modification of Open_Irr firmware is required, see the troubleshooting notes for additional software resources and considerations. The latest firmware will be available on the projects GitHub page [12].

**Fig. 14:**
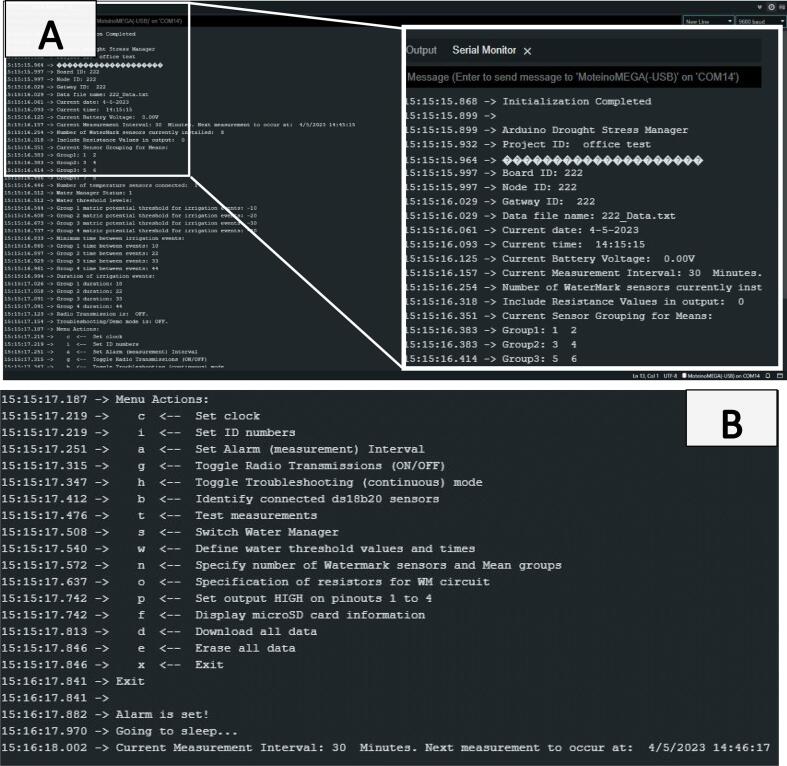
Alphabetically: (A) the main menu of an Open_Irr Node reports information pertaining to the settings of the Open_Irr Node for the end user. This menu is accessible using a Serial port monitoring application on a phone or computer connected to the Micro-USB port of the Moteino-Mega USB module of the Open_Irr Node. (B) The menu also serves as a user-interface for the device by providing a list of prompts that allow the end user to configure the Node as desired. Respond to a menu prompt by typing and sending the case sensitive letter into the Serial terminal via the Serial port monitoring application.

Note: user selection of any individual menu action must be made within 60 s, or the program will “timeout”, exiting the menu to operate under its current configuration. Menu actions are case-sensitive, use lower and uppercase letters where indicated. Menu functions describe how to configure the Open_Irr Node and Gateway devices in detail in Table 4 and Table 5, respectively. A simplified logic flow chart has been provided to aid end-user comprehension of the operation program of an Open_Irr Node (Fig. 15).

The Open_Irr system must be operated and configured through a device capable of Serial communication port monitoring e.g., computers, cell phones, and tablets. Computer users that have the Arduino IDE may utilize the Arduino program. Termite is a free alternative serial monitoring application available at several online repositories. For iPhone and Android, pick any reputable Serial monitoring application from the respective app store. In any Serial monitoring application, the baud rate must be set to 9600 and the user must select the communication port where the Open_Irr device is identified. Open_Irr devices become discoverable on a communication port automatically, the port number will be different between units but remain the same for an individual device. The Open_Irr device provides access to a configuration menu when a Serial port connection is established. The menu will appear as text in the Serial monitoring application utilized and the user will interface with the device by sending text commands in the Serial terminal monitoring application. There is no other user interface for configuration of the current Open_Irr system. The configuration menu reports the current values for most user-definable settings of the device. In addition to showing the current configuration, the menu provides the user with several prompts to configure the device. While the Open_Irr device is connected to a Serial terminal monitoring application and in troubleshooting mode, the user can type and enter “menu” into the Serial terminal monitoring application to return to the configuration menu. Menu options must be selected by typing and entering the desired case-sensitive command into the Serial port monitor terminal (Fig. 14).

**Table 4:** Open_Irr Node main menu options, functions, and a description of each function.

Menu selection	Function	Description
c	Set clock	Guides user through setting the time on the RTC module. Sequentially asks for user input of Month, Day, Year, Hour, Minute, and Second. Enter each of these as 2-digit numbers (i.e., for year 2022 type “22”).

i	Set ID numbers	Guides user through specifying a project ID, integer values attributed to the Node/Board ID and the GatewayID if a Gateway is utilized. The projectID may contain up to 32 characters and is included in the header of the datafile written to the MicroSD card. The Node/Board and Gateway IDs may be any 8-bit unsigned integer (0 to 255). The Node ID is present in each line of the datafile and uniquely identifies each Node while the Gateway ID indicates the destination of radio transmissions if radio transmission is enabled.

a	Set alarm (measurement) interval	Allows user to specify the sampling frequency for the Open_Irr Node in minutes. The Node will wake and perform 1 cycle through the main program loop before returning to a low-power sleep for the defined interval.

g	Toggle radio transmissions	Allows the user to disable radio transmissions in the event a Gateway unit is not utilized. Prompts the user to enter “1″ to enable radio transmissions or “0” to disable radio transmissions.

h	Toggle troubleshooting mode	Allows user to activate the troubleshooting mode to ignore the set measurement interval and continuously loop through the program. In troubleshooting mode, data is not saved to the microSD card. Radio transmissions and the water management system remains operational according to menu specifications “g” and “s”, respectively.

b	Identify connected DS18B20 sensors	Guides user through iterative identification of connected DS18B20 temperature sensors. The sensors have unique addresses which are called in the program. Upon first startup, and when adding or removing sensors, re-identify the connected sensors; start by unplugging ALL connected DS18B20 sensors. The system will ask you if you want to continue – if so type “1″, any other key will return the user to the start-up menu. The system will ask how many DS18B20 sensors are being connected, enter a number between 0 and 16. The user must then plug in individual sensors one-by-one and the system will identify the new sensor and save its address to the EEPROM memory of the Moteino-Mega. The order in which the sensors are plugged in is specified sequentially as 0 to 16 in the program and resulting datafile. When the specified number of DS18B20 sensors has been identified, the system will return to the startup menu.

t	Test measurements	The system will run through 1 cycle of the main program loop without saving or radio transmission of data. Used primarily to quickly check values of connected sensors.

s	Toggle water management system	Will ask the user if they want to enable the water management system of the Node; type 1 to enable and 0 to disable the water management system. When disabled, Open_Irr pinouts will not be set HIGH, regardless of water management group settings.When enabled, the Node will utilize current settings to determine if digital pins 12, 13, 14, and 3 of the Moteino-Mega (pinouts 1,2,3,4 left to right on the 4-pin terminal block, component 10 in Fig. 1) should be set HIGH. If not connected to irrigation infrastructure, the water management system should remain off.The water management system measures resistance (Ω) from all connected Watermark sensors, converts these values to soil matric potential (kPa) via manufacturer guidelines, and calculates a representative mean for each water management group (1 to 4) the user has defined in menu function “n”. The water management algorithm calculates a raw mean comparator for each water management group (1 to 4) . If the matric potential of any individual Watermark sensor is ≥ 20 percent different ($\frac{|V_{1}-V_{2}|}{\left[\frac{\left(V_{1}+V_{2}\right)}{2}\right]}\times 100$) from the comparator and ≥ ± 10 kPa from both the comparator and the user defined threshold level for an irrigation event (menu option “w”), that individual Watermark sensor is considered an outlier and dropped from the calculation of the representative group mean which is saved in the datafile. Note: no data is lost as readings from each individual sensor are also recorded. If the representative group mean kPa is ≤ (drier) the threshold specified for the group, that pinout (1 to 4) will be set HIGH for the time specified by the user in menu option “w”.

w	Specify water manager irrigation thresholds & details	Allows the user to configure irrigation events to be triggered by Open_Irr. First, the system asks the user which water management group (1 to 4) is being configured. Second, the system will show the current settings to reference for the group selected. Then, the system sequentially asks the user to enter: (i) the matric potential threshold, in kPa, that will prompt an irrigation event; (ii) the minimum time (in minutes) between successive irrigation events; (iii) the duration (in seconds) of the irrigation event. Finally, the system asks if inclusion of resistance measures (in Ω) should be included in the saved and radio transmitted data string if a Gateway is connected. Enter 1 to include or 0 to exclude these values from the written datafile and radio transmission.

n	Specify Watermark sensor details	User specification of the number of connected Watermark sensors and water management grouping (1 to 4) of sensors if desired. The system will prompt the user to enter 1 to continue; the user will be prompted to enter the number of installed Watermark sensors (0 to 16). Note: the sensors must be installed in sequential order of the respective 2-pin terminal block connectors on the PCB e.g., sensor 1 is connected to terminal block 1. Terminal block 0 may connect to a Watermark sensor but can be used to hold a fixed resistor to add a calibration factor to the algorithm converting resistance to matric potential. For additional information see the manufacturer’s website [14].After the number of Watermark sensors is defined the system will prompt the user for grouping instructions for group 1 to 4 sequentially. First, specify the total number (0 to 16) of Watermark sensors to include in the group; the number “0″ can be specified if no sensor grouping is desired. Second, enter the terminal block number (0 to 15) the sensor is connected to, for inclusion in the group until specifying the final sensor number in that group. The system will return to the startup menu where the user may check that the management group information was entered correctly.

o	Specify resistors	User must specify whether a calibration resistor, as mentioned in menu function “n”, is installed, enter “1” to indicate yes and “0” to indicate no. If a calibration resistor is included the user must specify the 2-pin terminal block (0 to 15) the calibration resistor is connected to. The user will be prompted to enter the value of the calibration resistor in Ω – we suggest a 10,000 Ω 1% tolerance resistor. Then, the user will be asked to enter the value of the fixed pull-up resistor (in Ω) that the analog reading is routed through in the Watermark reading circuit. This is resistor “R4” in the schematic, the source file circuit uses a 10,000 Ω resistor.

p	Sequentially set output HIGH on pinouts 1 to 4	Typically, only utilized when the water management system is active and irrigation infrastructure is connected to the Open_Irr unit. The user will be prompted to confirm and must enter “YES” to proceed. If confirmed, the system will sequentially (1 to 4) set the output HIGH on pins of the 4-pin terminal block for 10 s each. We use this function to prime irrigation lines or check ancillary irrigation infrastructure.

f	Display microSD card information	At the time of writing, a placeholder for a prompt to display information regarding allocation space on the connected microSD card. Non-functional at this time, the library function utilized does not report correct allocation information.

d	Print file data	The system will “print” all data stored on the microSD card under the current working filename to the serial monitor of the connected phone or laptop to allow a user to copy the data to another file. The filename will be “XXX_Data.txt” where X’s are replaced with the Node/Board ID or the Gateway ID when utilizing the gateway unit. Note: this can be an extremely long process if there is a lot of data on the microSD card. Consider the length of time the unit has been running and the sampling interval before selecting this prompt. It may be more efficient to turn the unit OFF, remove the microSD card and copy the file to an external device using a commercial microSD card reader.

e	Erase file data	Allows the user to erase the data on the microSD card saved under the current filename. The system will prompt the user to enter “YES” to confirm deletion of the file. There is no option to recover deleted data!

x	Save configuration & exit menu	**Important: selecting this option will save the current configuration settings to the EEPROM memory of the device.** Exits the startup menu and proceeds with operation. The startup menu will automatically “timeout” after 60 s if no action is specified.

**Table 5:** Open_Irr Gateway main menu selection options, their functions, and a description of each function.

Menu selection	Function	Description
b	Toggle Bluetooth serial interface	At the time of writing, a placeholder for a prompt allowing Bluetooth “classic” Serial port connection. Non-functional at this time. The Gateway is configured with a HC-05 Bluetooth module which will enable this interface option in the future.

c	Set clock	Identical to Node operation.

w	Define Gateway water management settings	The Open_Irr Gateway may be configured to set digital pins of the Moteino-Mega (19, 20, 21, 22) that connect, left to right, to the 4-pin terminal block connector on the Gateway to a HIGH state. The intended use case is to trigger irrigation events based on data received from connected Open_Irr Nodes. The user will be prompted to enter the water management group number they want to configure (1 to 4). Then, the user will be directed to a sub-menu to define settings for the selected setting number. Sub-menu options: “T”, Specify water management threshold levels. “E”, Set irrigation exclusion windows. “P”, Set irrigation permission windows. “G”, Define Node grouping details for Gateway water management group. “X”, Exit and return to main menu. “D”, Set duration of irrigation events (in minutes). “M”, Set the minimum time between irrigation events (in minutes). Note: all Nodes in the same water management group (1 to 4) should be configured with the same measurement/sampling frequency.

s	Toggle water management system	Identical to Node operation.

i	Set ID numbers	Identical to Node operation.

f	Display microSD card information	Identical to Node operation.

d	Print file data	Identical to Node operation.

e	Erase file data	Identical to Node operation.

l	Display LoRa radio settings	Prints current radio transceiver register settings. Parsing of the register values is intended for future firmware updates.

x	Exit menu	Identical to Node operation.

**Fig. 15:**
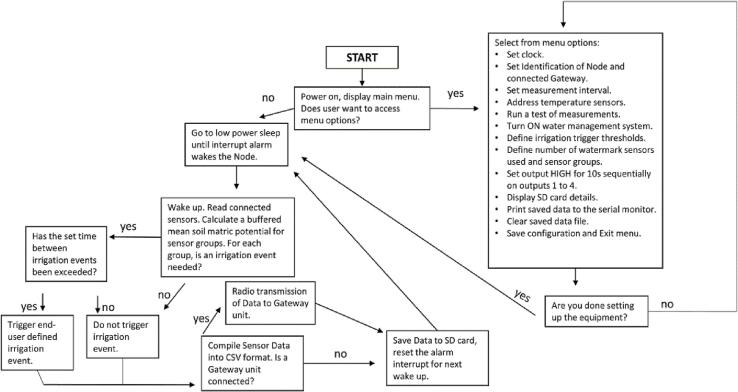
Logic flow-chart for an Open_Irr Node. Flow-chart depicting a simplified overview of the operation program running on an Open_Irr Node. Description of the main tasks an Open_Irr Node performs after user configuration are summarized.

When user configuration of an Open_Irr unit is completed, the user must select the “x” action in the menu to save the configuration changes to the devices EEPROM memory. Upon power cycle, Open_Irr devices populate a globally declared structure with the configuration from the EEPROM memory to call from during normal operation. It is our intention that users of Open_Irr devices will find the functionality limited but adequate through the current menu configuration options. Users with programming experience possessing other functionality requirements are encouraged to contribute to improving the Open_Irr platform through the device’s GitHub page [12].

Data from the Open_Irr system is stored on the MicroSD card in character separated value format using a comma “,” as the delimiter. A header describing the format of the saved data is provided in lines 1 to 8 of Open_Irr Nodes and 1 to 12 of Open_Irr Gateways with the delimited data beginning on line 9 and 13, respectively. On an Open_Irr Node the data is stored in the following order:

• Node ID
• Node battery pack voltage
• DateTime of the DS3231 RTC module
  o Month/day/year HH:MM:SS
• Watermark terminal block connector number (0 to 15)
  o Each connector number followed by the measured sensor value from that connector (kPa)
  o Each sensor value followed by the raw resistance value (Ω) if reporting of resistance values is specified in the configuration settings
• The water management group number (G1 to G4)
  o Each group number followed by the mean Watermark sensor value for the water management group
  o Each group mean followed by the DateTime of the last irrigation event for that group
    ▪ Month/day/year HH:MM:SS
• The DS18B20 temperature sensor number (0 to 15)
  o Each temperature sensor number followed by the value of the measurement in Celsius

On an Open_Irr Gateway the data from Node transmissions are stored in a similar fashion:

• Gateway ID
• Gateway battery pack voltage
• Node data string as described above

An example Open_Irr Node data string without raw resistance values: 104,7.92,4/2/2022 18:15:55,0,-58,1,-55,2,-58,3,-76,4,-44,5,-54,6,-45,7,-60,8,-74,9,-44,10,-54,11,-62,G1,-52,3/31/2022 9:32:13,G2,1,1/1/1970 0:00:00,G3,1,1/1/1970 0:00:00,G4,1,1/1/1970 0:00:00,T0,22.50,T1,23.19.

### General configuration of wiring

#### Sensors

**Fig. 16:**
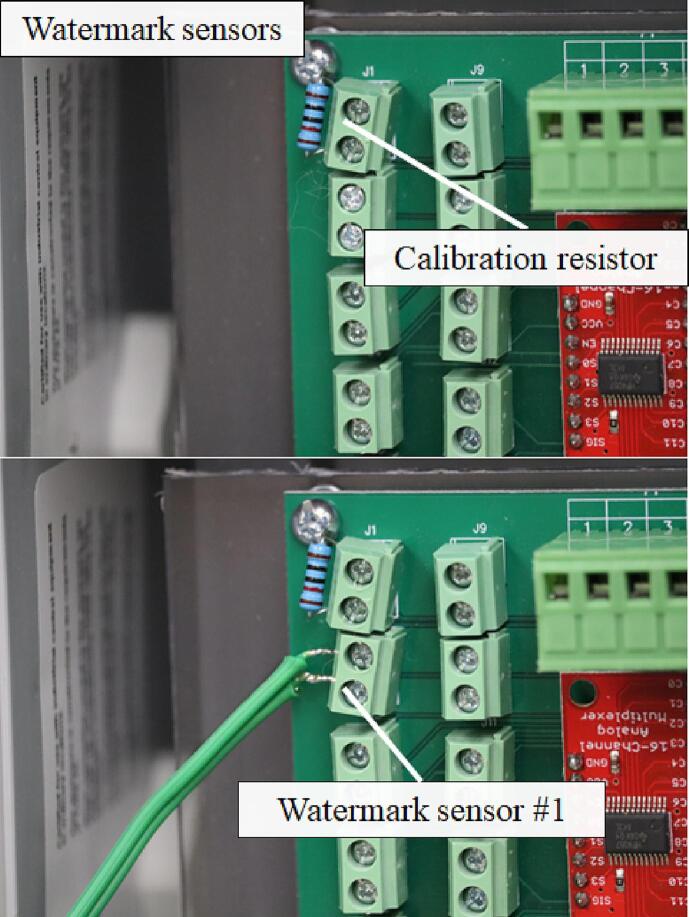
Watermark sensors may have either wire connected to each side of a 2-pin terminal block connector. Terminal block connectors are considered from 0 to 15 corresponding to multiplexor channels 0–15. An optional calibration resistor can be added to one of the terminal block connectors.

The Watermark sensors read by Open_Irr Nodes have two wires which are routed through a ¾-inch cable grommet to the 2-pin terminal block screw connectors, component 5 on the Node in Fig. 1. A calibration resistor may be placed in one of the 2-pin terminal block connectors if desired, this applies a calibration factor to the resistance readings from the Watermark sensors based upon the expected value of the calibration resistor. The presence, location, and value of the calibration resistor can be specified using menu option “o”. The reading program implemented by the Nodes excites and measures the analog Watermark sensors in each direction. Thus, either wire may be connected to one side of a single 2-pin terminal block connector (Fig. 16).

The DS18b20 temperature sensors are also wired through a ¾-inch cable grommet and into the 3-pin terminal block screw connectors, Node component 6 in Fig. 1. The wiring order for the temperature sensors from left to right must be: (i) Ground; (ii) Data; (iii) VCC (3.3 V from Moteino-Mega USB). Consulting the datasheet provided by the DS18b20 sensor vendor for identification of each wire is necessary as incorrect wiring of the DS18b20 temperature sensors may cause a short circuit and lead to device damage. To reduce the probability of occurrence, male–female 3-pin terminal block connectors have been specified (Fig. 4). Cable lengths may be increased for either sensor commensurate with manufacturer recommendations. For the Watermark sensors, total lengths ≤ 100 m are recommended; for the DS18b20 temperature sensors, total lengths ≤ 30 m are recommended.

#### Node and Gateway power

**Fig. 17:**
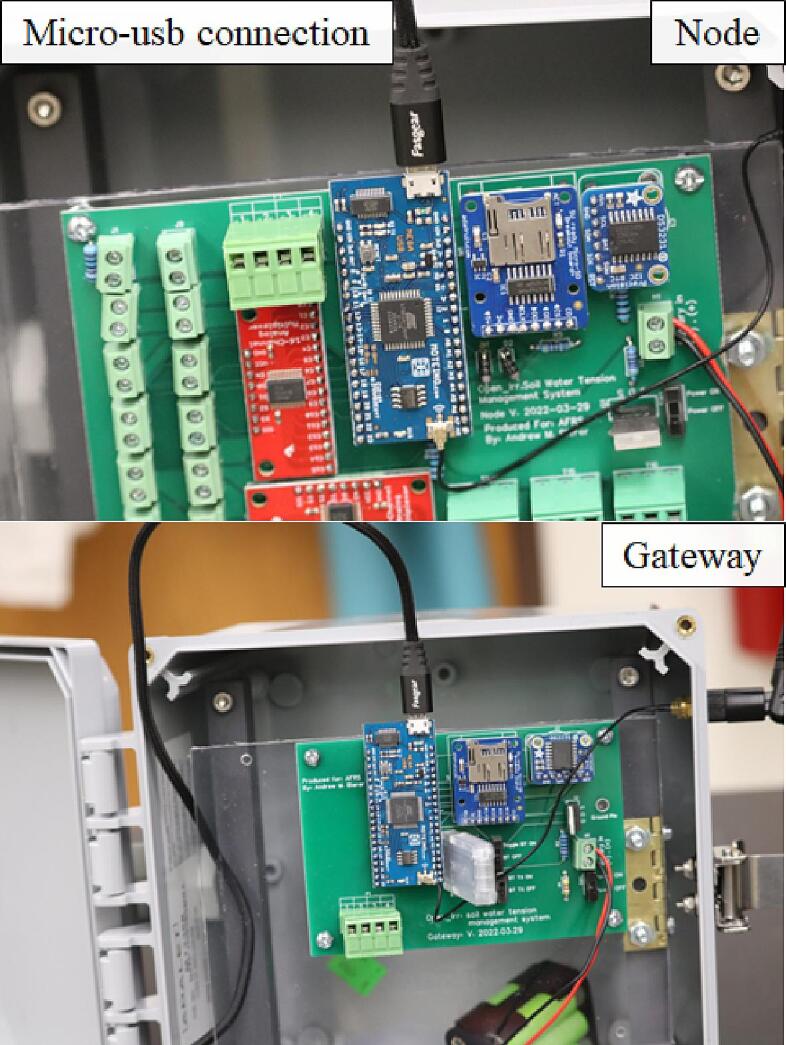
Micro-USB connection for user interface with the Open_Irr devices and an alternative power source.

Both the Node and gateway may be powered by a battery providing 3.3 to 16 V; a battery pack containing 6-AA NiMH batteries is specified for this manuscript. When convenient, power from a 3 to 16 VDC wall power supply can be provided to the Micro-USB port on the Moteino-Mega USB, however, note that the 3-pin power switch will not function to shut the unit off when this connection is made (Fig. 17).

For battery operation, connect the positive and negative wires from the battery cradle to the respective location on the 2-pin terminal block screw connector, Node component 9 in Fig. 1. When power supply sources are changes, i.e., plugging in a phone, tablet, or computer to the Micro-USB port of the Moteino-Mega USB, it is recommeded to first remove battery power via the 3-pin slide switch to minimize power spikes. In normal program operation, Open_Irr Nodes will return to a low-power sleep mode after completion of measurements; battery pack life span will be commensurate with enabling radio transmission or the water management routine, and the sampling/measurement interval. At a 15 min measurement interval, radio transmission and water management system enabled, and using six 2400 mAh rechargeable AA batteries, 1 to 2 weeks of Node operation can be expected. The Gateway may also be powered by the same six AA NiMH battery packs; however, connection to a permanent power supply is strongly recommended. The Gateway continually listens for incoming transmissions from connected Nodes and does not use a low-power sleep, thus requiring significantly more power. Therefore, a Gateway’s battery pack is best considered as a power supply back-up in the event of a black or brown out.

### Irrigation automation with Open_Irr

**Fig. 18:**
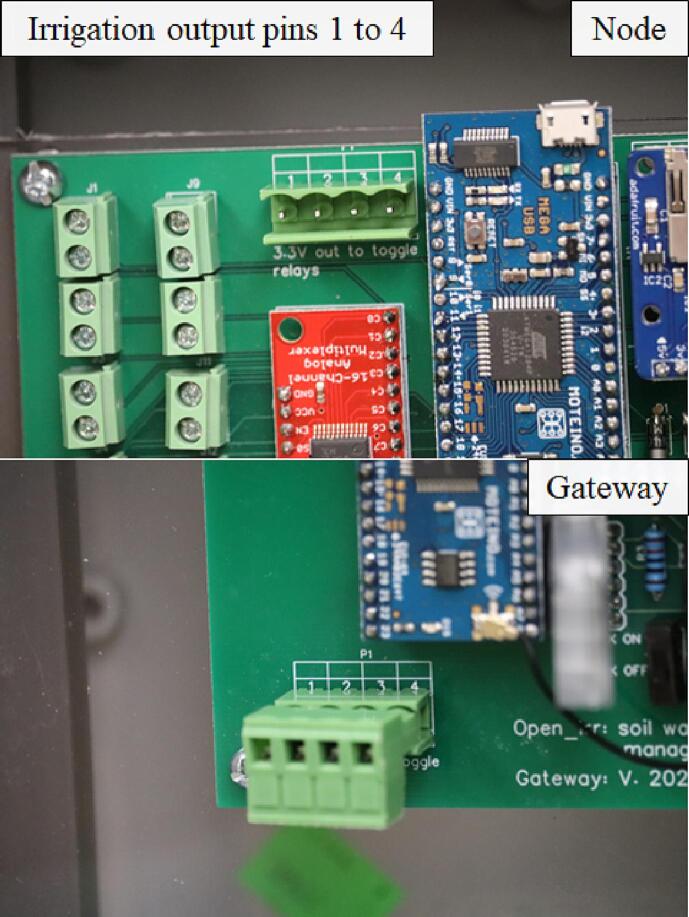
Pinouts 1 through 4 corresponding to water management groups 1 through 4 in Open_Irr devices. These pinouts can be used to output a 3.3 V signal based on Watermark sensor readings.

The Open_Irr platform allows for triggering of irrigation events based on sensor readings through a low-logic (3.3 V) HIGH output signal on up to 4 pinouts (Fig. 18).

**Fig. 19:**
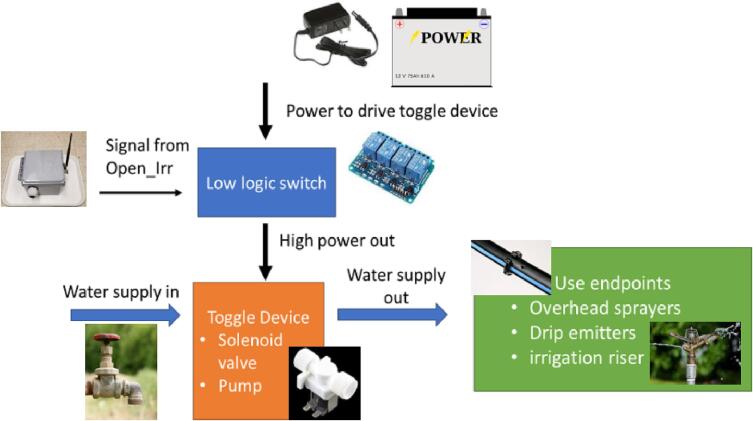
Use case diagram for irrigation automation with Open_Irr Diagram depicts required ancillary infrastructure for general automation of irrigation events. In addition to an Open_Irr device the following are required: (i) a low-logic capable switch device compatible with incoming power, a relay driver or MOSFET switch as examples; (ii) a toggle device compatible with incoming power, pumps and normally closed solenoids or ball valves as examples; (iii) a water supply which is routed to the toggle device, municipal spigot or a groundwater well, as examples; and (iv) a use end point and associated routing tubing, irrigation drip line and sprinklers, as examples.

Irrigation events on Nodes are based on connected sensor readings and defined using menu option “w”. On the Gateway, events are configured to occur based on sensor readings as received in radio transmissions from connected Nodes using menu option “w” and subsequently “G” as described in Table 5. The ability to send a low-logic (3.3 V) signal based on sensor readings is inherent to the Open_Irr devices; however, additional ancillary infrastructure is required for complete irrigation automation. A use-case diagram has been provided which provides a general description of the requirements (Fig. 19).

### Safe operation

The Open_Irr system may operate on battery voltages from 3.3 V to 16 V, exceeding this will damage the unit. We do not recommend attempts to interface higher-voltage systems while attempting to put together an automated irrigation system with Open_Irr if the user is unfamiliar with electrical wiring. Avoid electrostatic discharge to Open_Irr units as damage to system components can occur. Adding dehumidifying packets into the Open_Irr enclosure when operated in humid environments will reduce oxidation of system components. Do not power or operate Open_Irr units without an antenna connected as irreversible damage to the radio transceiver can occur. Note that we have described a 915 MHz radio transceiver as the Open_Irr platform was developed in the United States, please consult national radio laws in the country and region of operation before build and deployment [5]. Be careful not to bridge exposed microcontroller pins, short circuit can damage system components. Although unlikely, component failure can result in heat, smoke, and potentially fire. During operation, practice mindful cable management to avoid trip hazards. When wires must be routed along walkways, be sure to provide adequate warnings for passersby.

### Troubleshooting

• The Arduino IDE [18].
• Drivers for Moteino-Mega module used in Open_Irr [19].
• Patched version of the Radiohead library [20].
• Supporting documentation and latest firmware can be found at the Open_Irr Github page [12]. To contribute improvements to the Open_Irr platform: (i) fork the project and create a branch off the master to work in; (ii) make commits in this branch to improve the platform; (iii) push this branch to your forked master; (iv) open a pull request from the Open_Irr Github page; (v) discuss changes with others and make any required modifications; (vi) the project manager will merge or close the pull request. A handful of R scripts [21] for visualizing Open_Irr datafiles are available on the projects Github page.

## Validation and characterization

### Validation of accuracy and characterization of performance

**Fig. 20:**
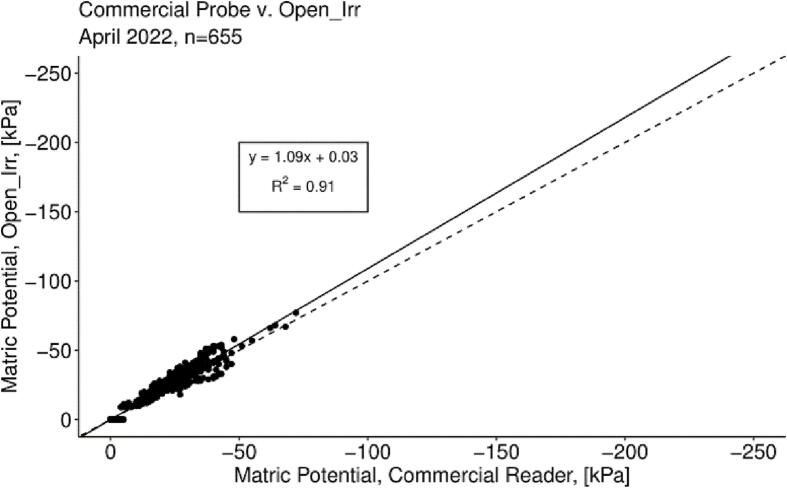
Soil matric potential, in kilopascals (kPa), recorded from Open_Irr Nodes as compared with values obtained with a commercial handheld Watermark sensor reading device. The solid line indicates the model fit while the segmented line indicates a slope of 1 and intercept of 0.

Soil matric potential measures obtained from the Open_Irr platform were assessed with those obtained from a commercial handheld Watermark sensor reader [8] sold by the sensor’s manufacturer. Data from the handheld device were recorded from 60 Watermark sensors deployed in potted apple trees between 9:00 and 10:00 am for 14 days during a greenhouse trial in April 2022. Open_Irr Nodes recorded data at a 15 min interval from the 60 sensors; data were matched with data from the handheld commercial reader by time using R [21], the *tidyverse* package [22] and its associated dependencies. Data were subset to remove instances of loose connections, incompatible time alignment, sensors having poor soil contact, and highly influential outliers identified using Cook’s distance criteria 4/n. A linear regression model was fit with the resulting reading pairs, n = 655, from each device (Fig. 20).

**Fig. 21:**
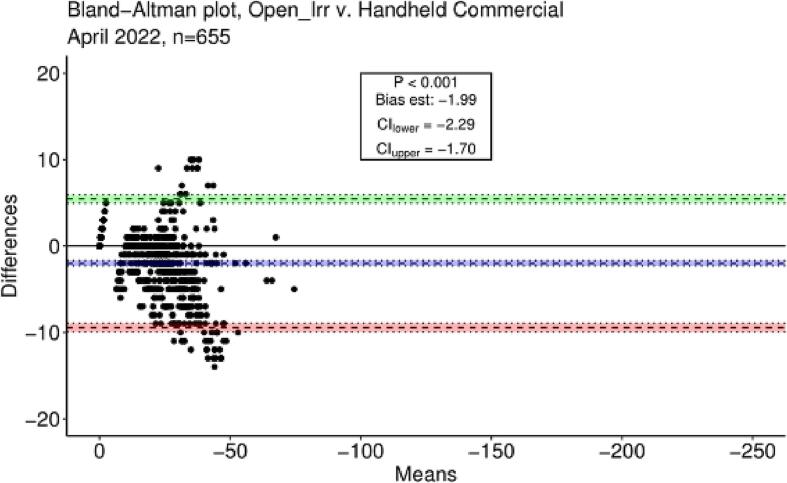
A Bland-Altman plot constructed from soil matric potential data recorded by Open_Irr Nodes and a handheld commercial Watermark sensor reading device. The green and red shaded regions indicate lower and upper limits of agreement and confidence intervals at the 0.05 probability level, respectively. The blue shaded region indicates the estimate of fixed bias and its confidence interval at the 0.05 probability level. (For interpretation of the references to colour in this figure legend, the reader is referred to the web version of this article.)

The estimated slope of the linear model was 1.09 with a standard error of 0.01; the intercept was estimated as 0.03 with a standard error of 0.33. In this case, a slope > 1 indicated slight overestimation of matric potential (more negative) by Open_Irr relative to the handheld commercial device. The coefficient of determination was 0.91 indicating high agreement between the two measurement devices. In methodological comparisons a slope converging on 1 is ideal, however systematic scatter around the regression may indicate potential biases. In response, a Bland-Altman plot was constructed utilizing the *blandr* package in R [23] by plotting the mean of the two measurement devices against the difference between means, permitting identification of any fixed bias or systematic differences between the Open_Irr Node and the handheld commercial reader [24] (Fig. 21).

The Bland-Altman estimate of fixed bias was −1.99 and considered significant at the 0.05 probability level suggesting soil matric potential values obtained through Open_Irr may be adjusted by 2 (less negative) to align with the commercial device. Practically, this difference may be nominal and originate from slight differences in the resistance conversion algorithm, such as temperature data source, inclusion of a calibration resistor, and miscellaneous resistance differences in the measurement circuits. Although not completely discernable in the range of matric potentials observed in this trial, continued formation of a funnel shape in the Bland-Altman figure at more negative matric potentials would suggest systematically increasing non-directional bias. Further study of the Open_Irr system at lower matric potentials in soils with higher clay content or experiments without live plants are needed to provide data for further speculation. Matric potentials at this extent were not explored in the preliminary trial as extreme water deficits would have manifested in tree mortality in the soil utilized.

**Fig. 22:**
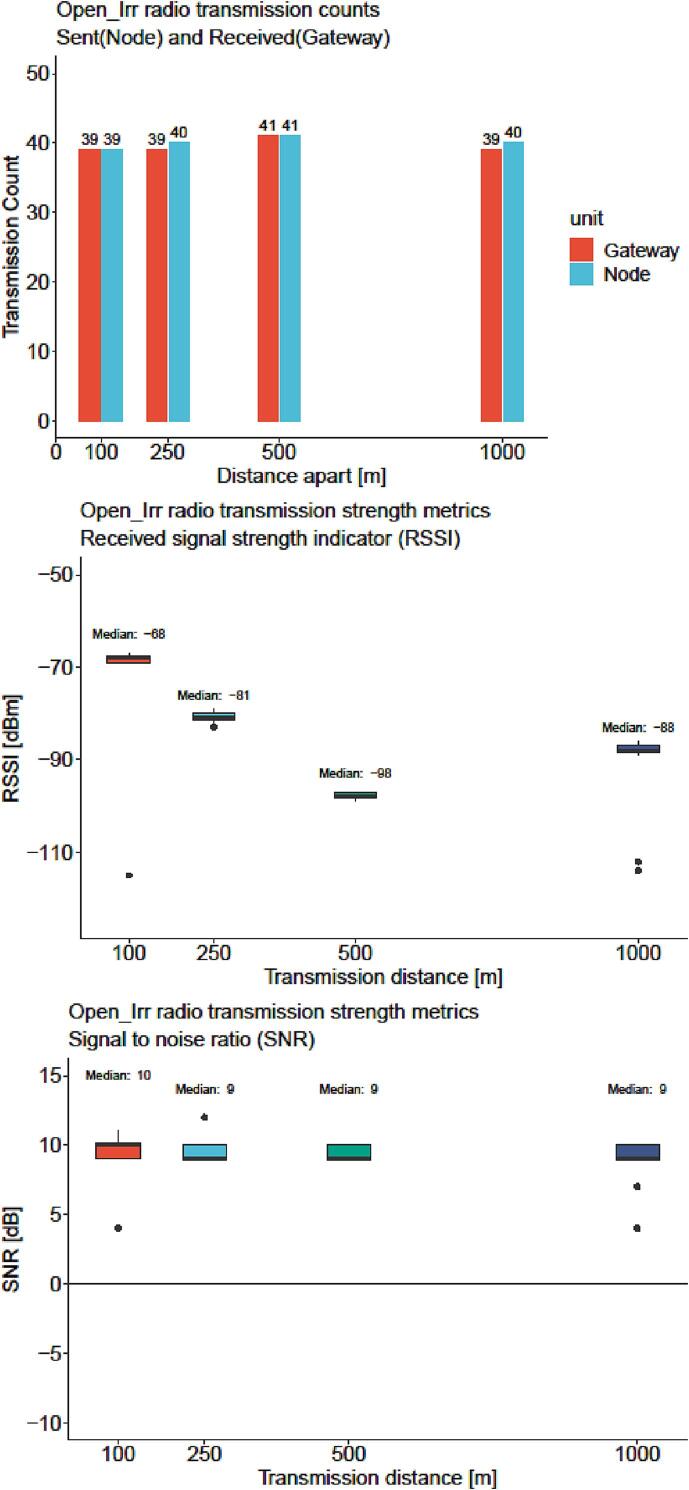
Results from radio transmission characterization of the Open_Irr Node and Gateway units. Expected v. received transmissions (top); boxplot of received signal strength indicators (middle) and signal to noise ratios (bottom) of units placed 100, 250, 500, and 1000 m apart.

A characterization of radio transmission reliability from Open_Irr Nodes to Gateways was completed. For this characterization, a single Node was configured for a measurement interval of 1 min with 15 Watermark sensors and 3 temperature sensors, then placed on a mast ∼ 1.5 m above the ground outdoors. The addressed Gateway was mounted on an identical mast and placed at 100, 250, 500, and 1000 m from the Node within line of sight. The elevation delta remained within ± 10 m at each distance. Weather at the time of testing was cloudy with temperatures remaining between 4 and 16 °C. Both Open_Irr units were powered on for 1 h at each distance. Note: although a measurement interval of 1 min was specified, the program run time and radio transmission function reduced the expected number of transmissions from 60 to approximately 40. Comparison between the transmissions expected from the Node and those received by the Gateway resulted in a ratio of 158/160 when distances were pooled, indicating a reliability of 99% (Fig. 22, top).

Two Node transmissions were not received by the Gateway, one at 250 m and one at 1000 m. Variability of the received signal strength indicator (RSSI) was low at each transmission distance, despite an occasional outlier, and was inversely related to transmission distance (Fig. 22, middle). Unexpectedly, the median RSSI was 10 dBm higher at 1000 m relative to 500 m. This may be attributed to landscape topography differences between the test sites. The 500 m test had to be conducted on a stretch of rolling hills; however, the landscape permitted a more direct line of sight in the 1000 m test from hilltop to hilltop. Nevertheless, at each distance RSSI remained above −100 dBm, above the −120 dBm minimum for LoRa communication. The signal to noise ratio (SNR) compares the received signal to the background noise in decibels. Observed median SNR values were 9 or 10 dB, far above the minimum of −20 dB for signal demodulation using the LoRa communication technique (Fig. 22, bottom). Results suggest that Open_Irr Gateways can reliably receive transmissions from Nodes ≤ 1 km away with direct line of sight. Obstructions such as buildings or trees and other devices operating within the same frequency band (915 MHz, USA) will interfere with radio transmissions and should be considered when placing Nodes and Gateways. This test should be considered representative of a real-world use case of the settings, antenna, and circuit of Open_Irr devices and not presumed to be representative of the holistic capability of the radio module utilized. For more information, readers are directed to the manufacturer of the radio module incorporated in the Moteino-Mega USB microcontroller [25].

### Demonstration of the Open_Irr hardware

**Fig. 23:**
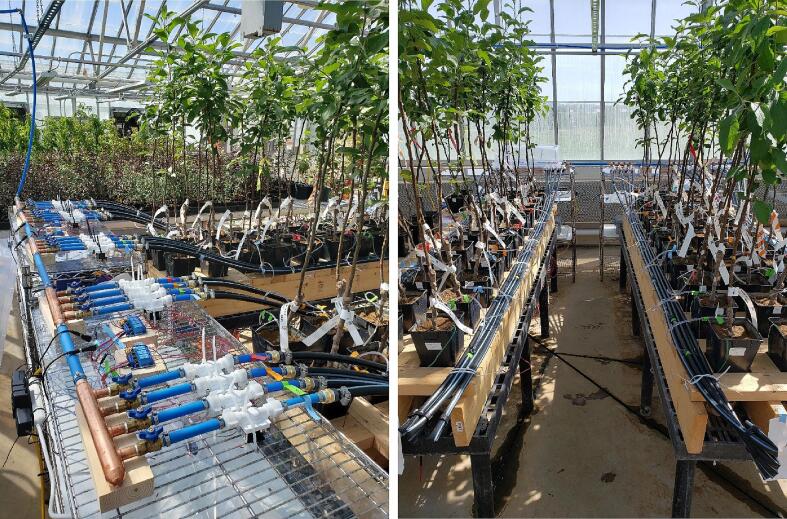
Deployment of the Open_Irr system and irrigation infrastructure in the preliminary trial. Photographs detailing equipment configuration for the preliminary trial of the Open_Irr system. Standard ½-inch PEX line distributed municipal water through a filter into an array of manifolds connected to manual shut off valves, ½-inch solenoid valves, and ¾-inch irrigation drip line. Relay modules were wired to control solenoid valves using negative logic.

The preliminary trial of the Open_Irr platform conducted in April of 2022 also intended to assess viability of automating irrigation events for purposes of horticultural water deficit study. Sixty apple (*Malus domestica* B.) B.9 rootstocks grafted with three scions (Autumn Gala, PP#12842; Crimson Crisp, PP#16622; Golden Delicious, Gibson Cultivar.) were planted in a climate-controlled greenhouse in pots containing Watermark and temperature sensors connected to Open_Irr Nodes. Five Nodes and 1 Gateway were deployed to monitor soil matric potential of the potted trees and automate irrigation events. From March to May, water management groups of the Nodes were configured to maintain unstressed conditions at −25 kPa to facilitate typical growth. On 16 May, the water management groups were configured to trigger irrigation events at −25, −40, −60, or −80 kPa corresponding to water management settings 1, 2, 3, and 4 of each Node. Other pertinent water management settings were: 15 min measurement frequency; (ii) a minimum of 15 min between irrigation events; and (iii) a 6 s irrigation event. To perform irrigation events, Open_Irr Node pinouts were wired to 4-channel 3.3 V logic relay modules [26] using negative logic configuration. In this configuration, VCC from a 12 VDC power supply was supplied to the toggle device through the relay module continuously, the relay closed the circuit upon receipt of the HIGH signal from Open_Irr Nodes by connecting the GND of the solenoid valve to the GND of the 12VDC power supply through the relay module. These relays were connected to ½-inch solenoid valves [27] and ¾-inch irrigation dripline installed with 0.5-gallon-per-hour emitters (Fig. 23).

**Fig. 24:**
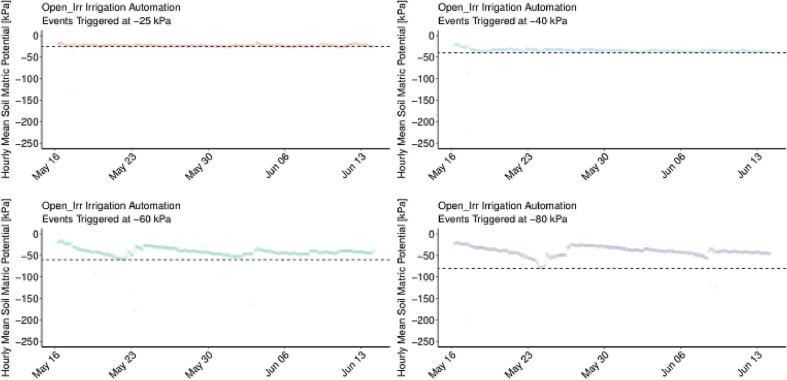
Soil matric potential measured by Open_Irr Nodes during implementation of four moisture content thresholds (-25 kPa, −40 kPa, −60 kPa, −80 kPa) to automate irrigation events. Prior to 16 May, a common threshold of −25 kPa was specified. Horizontal dashed lines indicate each moisture content threshold defined in Open_Irr water management groups, respectively. Point data are presented as hourly mean soil matric potential via density mapping to reduce clutter and improve interpretation.

It was observed that Open_Irr correctly triggered irrigation events at the defined matric potential thresholds; in the case of −60 or −80 kPa approximately 1 week had elapsed until irrigation events were triggered (Fig. 24).

**Fig. 25:**
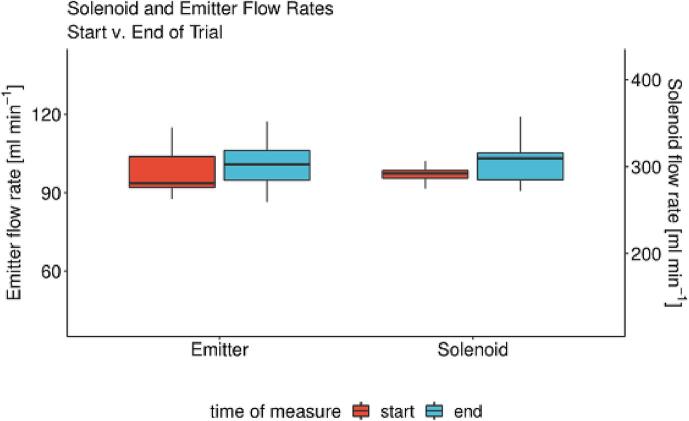
Flow rates, ml min^−1^, from individual emitters and solenoids as determined on 15 April and 14 June 2022, the start and end dates of the preliminary trial, respectively. Measurements were made using Open_Irr menu function “p”, a 10 s irrigation event, and repeated 3 times for each emitter. Flow rates for each emitter (12) per solenoid were pooled for comparison of solenoids.

The irrigation threshold of −40 kPa resulted in observable wilting of leaves, while −60 and −80 kPa resulted in partial and total desiccation, respectively, of foliage from 16 May to 23 May. At this point in time the Open_Irr water management group settings prompted irrigation events for −60 and −80 kPa thresholds, which recurred until the sensors ceased to read matric potential above respective thresholds. In this case, a lag-interval for sufficient re-watering and reduced water uptake manifested in matric potential spike and fall i.e., wet-dry cycles. Subsequent irrigation events for −60 and −80 kPa settings were delayed, likely due to the physiological stress encountered. The Open_Irr platform reliably triggered irrigation events at set points; however, proper selection of matric potential thresholds will maximize Open_Irr efficacy in tandem with modification of irrigation protocols for intended plant physiologic condition. Fine tuning of the water management algorithm is ongoing. As deployed in this preliminary study, Open_Irr Nodes opened solenoid valves for 6 s during a single irrigation event. To gauge the replicability of the experimental set up, the flow rate of individual emitters, and solenoid valves were measured at the outset and end of the study (Fig. 25).

Two sample t-tests indicated significant differences between the emitter and solenoid flow rates at the start and end of the trial, *P = 0.011 and P = 0.045* respectively. Numerically, confidence intervals constructed at the 0.05 probability level remained ± 8 and ± 26 ml min^−1^, respectively, and were considered suitable. Nevertheless, we recommend periodic calibration of any watering system connected to Open_Irr to assure accurate irrigation events, particularly for academic applications.

### Other considerations

To aid end-user deployment and operation of Open_Irr, several important observations were made while implementing the preliminary study. Initially, irrigation events were envisioned to be performed by peristaltic dosing micro pumps for the greatest accuracy and precision of irrigation events. The micro dosing pumps utilized were found to be unsuitable due to backpressure on the irrigation lines which prevented uniform irrigation events and prompted redesign of the irrigation system as described above. Second, as the study progressed the 3.3 V logic capable relay modules periodically failed; failure was traced back to individual channel relay switches and in a few cases to circuit isolation optocouplers on the relay switch boards. The failure of relay modules prompted daily oversight incompatible with commercial implementation. In response, an integrated power switching module has been developed and tested; incorporation of this module to the Open_Irr platform is planned for the next revision of the Open_Irr PCB. Last, radio transmission of long data strings as “String” type data from Open_Irr Nodes to the Gateway for reparsing was unreliable. Therefore, a custom library “RadioString” was created to better handle these situations and has been incorporated into the Open_Irr program. The “RadioString” library is also publicly available [28].

### Summary

To summarize, in fall of 2021 we initiated an endeavor to design a low-cost and open source datalogging platform to improve collection of soil water status and automate irrigation events for research and commercial applications in horticulture. The resulting Open_Irr system provides these functions at a competitive cost to end-users without requiring programming knowledge. Moreover, Open_Irr is a scale-neutral platform that provides end-users with the option to implement customized automated irrigation events. This function has the potential for widespread commercial applicability as demonstrated in a preliminary greenhouse trial using dripline irrigation and during the subsequent radio transmission range test. Moreover, the ability to specify the extremity of water deficit imposition is of further value to academic drought tolerance and cultivar screening endeavors for various crops. The authors hope that the Open_Irr platform will improve access to drought tolerance research tools, improve resiliency of current production systems and further the development of climate-smart and data-driven agricultural techniques.

### CRediT authorship contribution statement

**Andrew M. Bierer:** Conceptualization, Formal analysis, Investigation, Methodology, Validation, Visualization, Writing – original draft.

## Declaration of Competing Interest

The authors declare that they have no known competing financial interests or personal relationships that could have appeared to influence the work reported in this paper.
